# Relationship Between Temperaments of Herbal Diuretics and Their Effects Based on Avicenna’s Teaching

**Published:** 2017

**Authors:** Amir Mohammad Jaladat, Fatemeh Atarzadeh, Reihane Moeini, Ali Ghobadi, Omid Sadeghpour

**Affiliations:** a *Department of Traditional Medicine, School of Traditional Medicine, Shiraz University of Medical Sciences, Shiraz, Iran. *; b *Department of Traditional Medicine, School of Traditional Medicine, Tehran University of Medical Sciences, Tehran, Iran.*; c *Department of Traditional Pharmacy and Traditional Pharmacy & Medicine Research Center, Tehran University of Medical Sciences, Tehran, Iran. *; d *Herbal medicine department, research institute for Islamic and complementary medicine, Iran University of Medical Sciences.*

**Letter to editor**

Avicenna (980-1037 AD), the foremost Iranian physician, introduced numerous herbs as diuretics in his renowned book of* Canon of Medicine*. He has recommended herbal diuretics for most ailments accompanied by fluid superabundance like lassitude, oedema, and ascites. (1) 

He has classified diuretics as cold and hot ones according to their primary action and temperament (Table 1). Hot temperament diuretics have the power to dissolve or dilute viscous and congealed fluids from the pores of an organ and move them toward the urinary tract, while cold temperament diuretics act as a detergent and moistener to wash the residual matter from the body. (1)

Hot temperament diuretics are more potent than cold ones and can cause menorrhagia. They are contraindicated in the presence of irritative urinary symptoms whereas cold diuretics are advised for burning sensation secondary to urinary tract injuries and dryness. (1)

Today studies on the introduced herbal diuretics by Avicenna are scarce, though some have indicated their properties. For example Positive effects of cold temperament herbal diuretics like Pumpkin seeds in lower urinary tract symptoms of altered prostate health factors have been shown in clinical study (2) while hot diuretics like celery has been prohibited in the presence of symptomatic urinary tract infection due to its irritative volatile oil effect. (3)

The concept regarding potency of diuretic herbs, introduced by Avicenna is in line with the recent findings; hot temperament diuretics like parsley and celery are considered as strong diuretic herbs, (4) while cold ones like *Malvaceae* are used as mild diuretic. (5) 

In recent investigation hot temperament diuretic herbs like anise, and fennel that are dissolvent were more efficient in decreasing weight of normal rats than cold ones such as cucumber, watermelon, and pumpkin; however, none of them did not increase the amount of urine output significantly. (6) 

Overall, it seems that different medicinal indications could be considered for hot and cold temperament herbal diuretics, they do not act specifically in the nephrons, and they may play different therapeutic role in gastrointestinal and urogenital systems.

**Table 1:** Different classes and actions of herbal diuretics in the* Canon of Medicine*. (1, 7)

**Common name**	**Scientific name**	**Name in ** ***Canon***	**Part used**	**family**
**Common hot temperament / strong diuretics** **(Act as d** **issolvent, deobstruent, and emmenagogue)**				
Anise	*Pimpinella anisum*	Anisun	Fruit	Apiaceae
Black seed	*Nigella sativa*	Shuniz	Fruit	Ranunculaceae
Celery seed	*Apium graveolens*	Karafs	Fruit	Apiaceae
Carrot fruit	*Daucus carota*	Jazar	Fruit	Apiaceae
Cassia bark	*Cinnamomum cassia*	Salikhah	Bark	Lauraceae
Fennel	Foeniculum vulgare	Razianeh	*Fruit*	Apiaceae
Orris root	*Iris germanica*	Irsa	Rhizome	Iridaceae
Asparagus	*Asparagus racemosus *Willd	Hilyun	Root	Asparagaceae
Parsley	*Petroselinum crispum* (Mill) Fuss	Futrasaliyun	Whole plant	Apiaceae
**Common cold temperament / mild diuretics** **(Act as a detergent ** **and moistener** ** and useful in lower urinary tract symptoms like dysuria)**				
Cucumber	*Cucumis flexuosus*	Qitha	Fruit	Cucurbitaceae
Pumpkin	*Cucurbita pepo*	Qar	Fruit	Cucurbitaceae
Common malva	*Malva sylvestris*	Khubbazi	Flower	malvaceae
Purslane	*Portulaca oleracea*	Baqla hamqa	Leaves and seeds	Portulacaceae
Small caltrops	*Tribulus terrestris*	Hasak	Fruit	Zygophyllaceae
Winter – cherry	*Physalis Alkekengi*	Kakenj	Fruit	solanaceae
